# Hemin-Induced Transient Senescence Via DNA Damage Response: A Neuroprotective Mechanism Against Ferroptosis in Intracerebral Hemorrhage

**DOI:** 10.21203/rs.3.rs-4686841/v1

**Published:** 2024-07-26

**Authors:** Vikas H. Maloji Rao, Velmarini Vasquez, Manohar Kodavati, Joy Mitra, Vincent Provasek, Anh Voh, Anton Liopo, Paul J. Derry, Andrei Mikheve, Robert C. Rostomily, Philip J. Horner, James M. Tour, Gavin W. Britz, Thomas A. Kent, Muralidhar Hegde

**Affiliations:** 1 Division of DNA Repair Research within the, Department of Neurosurgery, Houston Methodist Research Institute, Houston, TX 77030, USA; 2 Center for Neuroregeneration, Department of Neurosurgery, Houston Methodist Research Institute, Houston, TX 77030, USA.; 3 Center for Genomics and Precision Medicine, Department of Translational Medical Sciences, Institute of Biosciences and Technology, College of Medicine, Texas A&M Health Science Center, Houston, TX 77030, USA.; 4 Department of Neuroscience, Weill Cornell Medical College, New York, NY 10065, USA.; 5 NanoCarbon Center and the Rice Institute for Advanced Materials, Department of Chemistry, Rice University, Houston, TX 77030, USA.; 6 Stanley Appel Department of Neurology and Department of Radiology, Houston Methodist Institute of Academic Medicine and Research Institute, Houston, TX 77030, USA.; 7 Department of Chemistry, Rice University, Houston, TX 77005, USA.

**Keywords:** Hemorrhage, Senescence, DNA damage response (DDR), Heme oxygenase-1 (HO-1), Ferroptosis, Adaptive neuroprotection

## Abstract

Intracerebral hemorrhage (ICH) poses acute fatality and long-term neurological risks due to hemin and iron accumulation from hemoglobin breakdown. Our observation that hemin induces DNA double-strand breaks (DSBs), prompting a senescence-like phenotype in neurons, necessitating deeper exploration of cellular responses. Using experimental ICH models and human ICH patient tissue, we elucidate hemin-mediated DNA damage response (DDR) inducing transient senescence and delayed expression of heme oxygenase (HO-1). HO-1 co-localizes with senescence-associated β-Galactosidase (SA-β-Gal) in ICH patient tissues, emphasizing clinical relevance of inducible HO-1 expression in senescent cells. We reveal a reversible senescence state protective against acute cell death by hemin, while repeat exposure leads to long-lasting senescence. Inhibiting early senescence expression increases cell death, supporting the protective role of senescence against hemin toxicity. Hemin-induced senescence is attenuated by a pleiotropic carbon nanoparticle that is a catalytic mimic of superoxide dismutase, but this treatment increased lipid peroxidation, consistent with ferroptosis from hemin breakdown released iron. When coupled with iron chelator deferoxamine (DEF), the nanoparticle reduces hemin-induced senescence and upregulates factors protecting against ferroptosis. Our study suggests transient senescence induced by DDR as an early potential neuroprotective mechanism in ICH, but the risk or iron-related toxicity supports a multi-pronged therapeutic approach.

## Introduction

Intracerebral brain hemorrhage (ICH) is a life-threatening medical condition affecting over 15 million people worldwide annually, with many poor outcomes despite recent treatment advances^1^. Hemorrhages account for about 40% of stroke-related mortality and have poor prognosis^2, 3^. Although TBI is not classified as hemorrhage, it frequently involves intracranial bleeding^4^. Current treatment guidelines recommend a comprehensive medical approach involving intensive management of coagulopathy and blood pressure, as well as strategies to prevent secondary brain injury and control intracranial pressure. However, medical interventions often fall short, with approximately 40% of patients dying within 30 days of an ICH, and around 80% of survivors experiencing significant disabilities While recent studies show significant functional improvement from surgical clot evacuation interventions, finding definitive long-term treatment options remain elusive^5,6,7,8^. Macro hemorrhages and cerebral microbleeds reportedly increase by nearly 2-fold the risk of developing neurodegenerative disorders^9^. Global burden of disease studies finds the number of ICH has increased by 47% from 2000 and 2010 and appears to disproportionally affect ICH mortality in low-to-middle-income countries^7^. Hemorrhage can cause primary and secondary brain damage, with the former resulting in mechanical injury to the brain due to the abrupt buildup of leaked blood in an enclosed space, the clavarium^10^. Secondary damage is likely caused by blood-derived products, including hemin and iron not typically encountered by brain tissues. Ours and others prior work implicates these factors contributing to cellular dysfunction post-ICH^7,8^. The secondary damage can occur from hours to days after the primary brain damage, resulting in progressive changes in cellular and molecular processes ^11^. Depending on the size of the hematoma, cells in the proximity may be exposed to as much as 10 mM concentrations of hemin and iron^12, 13^. However, the underlying mechanisms behind toxicity induced by hemin and free iron are not completely understood, presenting a major challenge in the clinical management of the chronic effects of these conditions. Although inflammation, reactive oxygen species (ROS), and excess redox iron have been shown to play critical roles in hemorrhage-induced neurotoxicity, therapeutic efforts based solely on antioxidants or iron chelators have not been definitively effective in late-stage clinical trials^14.^ Therefore, a re-evaluation of mechanisms to guide the development of new therapeutic approaches is necessary.

Hemin is a highly reactive free radical and pro-oxidant^15^. It is degraded by the inducible isoform of the heme oxygenase (HO) enzyme, known as HO-1, which is inducible and activated upon exposure to hemin both *in vitro* and in ICH patient brain^16, 17^. HO-1 degrades hemin into biliverdin, carbon monoxide, and free iron (Fe^2+^)^18^. Additionally, HO-1 exhibits anti-inflammatory and antioxidant properties, contributing to its neuroprotective effects^19^. If expressed in a timely and sufficient manner, particularly in non-threatening mild hemorrhagic conditions, HO-1 can modulate inflammatory pathways, diminishing the release of pro-inflammatory cytokines, thereby limiting tissue damage, and promoting repair^20^. In contrast, HO-2, which is constitutively expressed in neurons, to carry out basal heme oxygenase activity, is not induced by hemin exposure^21, 22^.

Iron, at physiological levels, is an essential metal, involved in oxygen transport (hemoglobin), electron transport within the mitochondria, important redox reactions, and a factor in nucleotide synthesis for cell division and myelination. Iron homeostasis is regulated. Its dysregulation has been associated with neurodegeneration^4, 23^.

We recently reported that hemin rapidly induces DNA double-strand breaks (DSBs) in the genomes of neurons and endothelial cells, as well as in mouse brains following experimental ICH induction^24^. This was accompanied by the activation of robust DNA damage response (DDR) signaling and the expression of a senescence-like phenotype. Senescence is known to inhibit other cell death pathways including iron-mediated ferroptosis ^25^, potentially providing protection against the elevated iron levels associated with ICH^26^. However, persistent senescence can lead to a detrimental inflammatory phenotype ^25^. Investigating these mechanisms is crucial for a deeper understanding of the protective versus pathological roles of complex cellular responses to hemin and iron toxicity. Such insights are essential for developing effective strategies to mitigate the long-term neurological effects of blood toxicity.

In this study, we present evidence that hemin-induced DDR is a direct trigger for an early senescence-like phenotype, characterized by β-Galactosidase (β-Gal) positivity in cells. DDR is also critical for inducing the expression of HO-1, predominantly in β-Gal positive cells. We find a sequential relationship starting with hemin-induced DSBs, which trigger β-Gal, culminating in the selective expression of HO-1 in these cells. We observed a transient and reversible senescence state under a single-moderate hemin exposure, which becomes long-lasting upon repeat hemin exposure. Inhibiting this early senescence expression by blocking DDR or p21, surprisingly increased cell death characterized by increased lipid peroxidation, an endpoint associated with ferroptosis, suggesting that transient senescence may offer protection against excess iron^26, 27^. Notably, inhibiting senescence using a pleiotropic oxidized carbon nanoparticle, with iron chelator deferoxamine (DEF), counteracts both hemin-induced senescence and lipid peroxidation, an endpoint associated with ferroptosis, in cultured neurons. Our study highlights the significance of DDR-dependent transient senescence as a potential adaptive mechanism for neuroprotection in this in-vitro model of ICH, demonstrated by the therapeutic, neuroprotective advantage of not interfering with transient DDR-dependent senescence while inhibiting hemin and chelating iron. These insights mark a significant advance in our understanding of sequential hemin-mediated expression of DDR, senescence, and HO-1, and may pave the way for therapeutic approaches targeting the complex interplay between DDR, senescence, and hemin/iron toxicity in ICH.

## Materials and Methods

### Antibodies, kits, and cell culture reagents

The primary antibodies used in the study were obtained from various sources, including Abcam, Cell Signaling Technology, R&D system, Sigma-Aldrich, and GeneTex, as detailed below: p-ATM (ab81292; 1:1000), ATM (ab47575; 1:1000), p-53BP1 (CST2675; 1:1000), 53BP1 (CST4937; 1:1000), γH2AX (CST9718; 1:1000), Histone H2AII (CST2578; 1:1000), p-NF-KB(CST3033; 1:1000), Cleaved PARP (CST9546; 1:1000), Caspase-3 (CST9665; 1:1000), glutathione peroxidase 4 (CST52455; 1:1000), p21 (CST2947; 1:1000), NRF2 (CST12721; 1:1000), HO-1 (AF3776; 1:1000), HO-2 (MAB3170; 1:1000), p53 (SAB5700817; 1:1000), and β-actin (GTX109639; 1:4000). KU 55933 (#3544) and Pifithrin-α (# 3843) were purchased from Tocris (Bristol, UK). UC2288 (#5328130001), BMS-345541(#B9935), ML385 (#SML1833), and HO-1 siRNA were purchased from Millipore Sigma (Burlington, MA, USA). The SA-Beta-Galactosidase staining kit (CST#9860) was purchased from Cell Signaling Technology, and hemin (#51280) and all-trans retinoic acid (#R2625) were purchased from Sigma. DNeasy blood and tissue kit (#69504) were purchased from Qiagen. Cell imaging slides (#PEZGS0816) were purchased from Millicell EZ (EMD Millipore, Darmstadt, Germany). Click-IT lipid peroxidation imaging kit (Cat. No. C10446), Cell culture reagents such as Dulbecco’s modified Eagle/F12 medium (DMEM/F12), trypsin-EDTA, fetal bovine serum (FBS), Tryphan Blue and penicillin/streptomycin were purchased from Gibco BRL (Thermo Fisher Scientific). HO-1 plasmid (pCX-HO1–2A-EGFP) was purchased from Addgene (plasmid # 74672).

### Neuronal culture, differentiation, and inhibitor treatment

Human induced pluripotent stem cell (iPSC)-derived neural progenitor cells (NPSCs). The culture and differentiation of human NPSCs were performed according to a protocol published by Vasquez et. al^28^. The NPSCs were generated from the control human iPSC line KYOU-DXR0109B (201B7, ATCC). The cells were plated at 9.0 × 105 cells per 6 cm2 dish coated with 20μg/mL Poly-L-ornithine (Sigma) and 10 μg/mL laminin (Gibco, 23017–015) and cultured with the StemPro NSC SFM media (Gibco, A10509–01) with 5 μM Rock Inhibitor (ATCC) and dissociated using accutase when confluent. For differentiation, the media was replaced with neural induction media (NIM) consisting of neurobasal media (Gibco), 2% B-27 supplement (Gibco), and 1X GlutaMAX-I Supplement (Gibco). The NIM was replaced every two days, and at day seven of differentiation, it was supplemented with 0.5 mM of dibutyryl cAMP (Sigma) and further maintained for five days.

HBEC-5i and SH-SY5Y lines. The SH-SY5Y (CRL-2266) and HBEC-5i cell lines (CRL-3245) were purchased from ATCC (Manassas, VA, USA). HBEC-5i cells were cultured in DMEM-F12(1:1), supplemented with 10% FBS, 1% P/S, and 40 μg/mL endothelial growth supplement (ECGS) (Sigma E2759). The SH-SY5Y human neuroblastoma cell line was cultured in DMEM/F12 (1:1) supplemented with 1% FBS, 1% P/S, and differentiated with medium containing 0.5% FBS and 10 μM all-trans retinoic acid (RA) for 4 days.

Pharmacological inhibitors, including KU-55933 (ATMi), Pifithrin-α (p53i), UC2288 (p21i), BMS-345541 (NF-KBi), and ML385 (NRF2i), were pretreated to the experimental group with or without hemin. The inhibitors were prepared at 1 mM stock solutions in DMSO and then diluted in DMEM/F12 media with 0.5% FBS. All the inhibitors used in the current study were prepared at 1 mM stock solution in DMSO. The time points and concentrations of the inhibitors were indicated and compared with the control DMSO.

### Hemin preparation and treatment

A 1 mM stock solution of hemin was prepared by dissolving in DMSO. To prepare the working solution, the stock was diluted with DMEM/F12 media (0.5% FBS) to a final concentration of 10 μM, which was then vortexed and immediately used for *in vitro* treatment. A new stock solution was prepared each time the experiment was performed.

### Plasmids, siRNA, and transfection

The GFP-tagged HMOX1 plasmid (pCX-HO1–2A-EGFP) was generously provided by Roberto Giovannoni (Addgene plasmid # 74672^29^). Plasmid transfection using Lipofectamine 3000 was conducted to establish the stable expression of HO-1 in SH-SY5Y cells. RNA interference was utilized, employing negative control small interfering RNA (NC-siRNA) and HO-1 siRNA, both acquired from Millipore Sigma (Burlington, MA, USA). The specific sequence of the HO-1 siRNA that was used was ATATTCTCCCAGGAGTACA. To initiate the transfection process, a mixture containing HO-1 siRNA, Opti-MEM (Gibco, Grand Island, NY, USA), and Lipofectamine 3000 (Invitrogen, Groningen, Netherlands) was incubated at room temperature for 15 min. Subsequently, the SH-SY5Y cells were subjected to reverse transfection and then incubated at 37°C in a CO2 incubator for 24 h, preparing them for the gene knockdown assay.

### PEG-OAC and DEF-OAC-PEG nanoparticles

PEG-OACs are a multifunctional nanozyme that demonstrate catalytic superoxide dismutase mimetic properties, quench the hydroxyl radical, protect mitochondria through supporting electron transfer and catalyze the oxidation of hydrogen sulfide to protective polysulfides. They are the latest generation of a series of oxidized carbon nanoparticles synthesized by our labs that have been shown to be protective *in vivo* and *ex vivo* in a variety of models^30^. The synthesis of PEG-OAC and DEF-OAC-PEG nanoparticles was conducted following the procedure outlined by Dharmalingam and McHugh et al^24, 31^, employing medical-grade coconut-shell activated charcoal (AC). To produce uniform 3–8 nm particles with transformed electron transfer properties, AC was oxidized using fuming nitric acid. Subsequently, oxidized AC (OAC) was covalently bound through carbodiimide coupling to poly(ethylene)-glycol (PEG; PEG-OAC). To synthesize PEG-OAC with Deferoxamine (DEF) a 50:50 mix of DEF and PEG were subjected to the same carbodiimide coupling reaction, generating DEF-OAC-PEG nanoparticles^23^.

Both PEG-OAC and DEF-OAC-PEG nanoparticles were stored in glass vials at room temperature and shielded from light exposure. The DEF-OAC-PEG vials were additionally wrapped in aluminum foil to protect Deferoxamine, as it is sensitive to light. To minimize air oxidation, all the vials were purged of oxygen and replaced with argon.

### Cell viability, cytotoxicity, and caspase activation analysis

ApoTox- Glo Triplex Assay (Promega, Madison, WI, USA) was used to determine viability, cytotoxicity, and apoptosis. The assay evaluates different cellular events by measuring live-cell protease activity using a fluorogenic, cell-permeant peptide substrate (GF-AFC), dead-cell protease activity using a cell-impermeant, fluorogenic peptide substrate (bis-AAF-R110), and caspase-3/7 activation is measured by luminogenic DEVD-peptide substrate. The cells were seeded in 96-well black clear bottom plates, and the assay was carried out according to the manufacturer’s instructions.

### CellTiter-Glo 2.0 cell viability assay

Cells were cultured in a white opaque 96-well plate. After subjecting the cells to varying culturing condition for the specified duration, the plate, along with its contents, was brought to room temperature for about 30 minutes. Subsequently, an amount of CellTiter-Glo 2.0 reagent (Promega-G9242), equivalent to the volume of the cell culture medium in each well, was added. The mixture was then agitated for 2 minutes on an orbital shaker to promote cell lysis, followed by a 10-minute incubation at room temperature to allow the luminescent signal to stabilize. The luminescence was measured using a Tecan microplate reader. This luminescent signal correlates with the ATP level in the sample, serving as an indicator of the presence of viable and metabolically active cells.

### Trypan blue assay

The trypan blue assay protocol was followed as described by Leo Li-Ying et al^32^. After subjecting the cells to the treatment condition, they were rinsed with PBS and trypsinized. Next, a 20 μl cell suspension was combined with 20 μl of trypan blue stain, allowing for the enumeration of live and dead cells using a Countess 3 Automated Cell Counter.

### Immunofluorescence (IF) assay

Cells were cultured in cell imaging chamber slides until they reached 70% confluence and then subjected to different experimental conditions. Cells were then fixed, permeabilized, and incubated with the desired primary antibody in 1% BSA/PBS overnight at 4 °C, followed by incubation with fluorophore-conjugated secondary antibody. Finally, the DAPI-counterstained images were captured using an AXIO Observer inverted microscope olympus confocal microscope fv3000 (Shinjuku City, Tokyo, Japan).

### IF staining for lipid peroxidation

Intracellular lipid peroxidation was assessed using the Click-IT lipid peroxidation imaging kit (Cat. No. C10446; Invitrogen, Waltham, MA, USA) as per the manufacturer’s guidelines. Briefly, cells were seeded on chamber slides at a density of 4 × 104 cells per well. After the specific treatment conditions, the cells were fixed with 4% paraformaldehyde, permeabilized using 0.1% Triton-X100, and blocked with 1% bovine serum albumin (BSA) for 30 min at room temperature. Subsequently, a Click-IT reaction mixture, containing reaction buffer, CuSO4, Alexa Fluor 488, and additive buffer, was applied and incubated for 30 min at room temperature. The cells were then washed twice with 1% BSA and twice with PBS. Finally, cellular nuclei were stained with DAPI, the cells were visualized, and images were acquired using an EVOS fluorescence microscope.

### SA-β-Gal activity

The SA-β-Gal activity assay was performed according to the manufacturer’s protocol (#9860; Cell Signaling). Briefly, cells were grown on a 35 mm plate and exposed to different experimental conditions. The cells were fixed in a solution containing 2% formaldehyde and 2% glutaraldehyde for 10 min and stained overnight at 37°C in a dry incubator using a staining solution (X-Gal substrate) at pH 6.0 for 15 h. Approximately 500 cells from each treatment group were analyzed using a light microscope. Images were acquired using EVOS^™^ FL Auto Imaging System.

In addition, the CellEvent^™^ Senescence Green Detection Kit (Thermo Fisher Scientific) was used to identify senescence cells by fluorescence. After treatment with different conditions, cells were fixed with 4% paraformaldehyde and treated with a prewarmed X-Gal substrate solution for 2 h at 37°C in the absence of CO2, following the manufacturer’s instructions. After incubation, the cells were permeabilized and then stained with primary antibodies. The images were captured using AXIO Observer inverted microscope (Carl Zeiss, Oberkochen, Germany).

### Single-cell gel electrophoresis (Comet) assay

The neutral comet assay was performed on cells before and after they were treated with hemin, according to the manufacturer’s protocol (Trevigen, Gaithersburg, MD, USA). Briefly, cells from different experimental groups were mixed with low-melting grade agarose and placed on comet assay slides (also provided by Trevigen). The slides were then incubated in a lysis buffer, followed by immersion in a neutral electrophoresis buffer. The slides were subjected to electrophoresis at 21 V for 45 min. Next, the slides were fixed with 70% ethanol and stained with SYBR Green for visualization using a fluorescence microscope (EVOS^™^ FL Auto Imaging System). The comet tail moment was scored using the Open Comet plugin for ImageJ software on 50 randomly selected cells.

### Long-amplicon polymerase chain reaction (LA-PCR)

The genomic DNA from different experimental groups was extracted using the DNeasy Blood and Tissue kit (Qiagen), per the manufactureŕs instructions. To assess genome integrity, the LongAmpTaq DNA polymerase (New England Biolabs, Ipswich, MA) was used to amplify a ~10.9 kb genomic DNA region (HPRT gene, exons 2–5, Accession number J00205) with specific primers (forward: 5’ TGGGAT TACACGTGTGAACCAACC-3’; reverse; 5’ GCTCTACCCTGTCCTCTACCGTCC-3’)^33^. Additionally, a control, a shorter fragment of ~250 bp, was also amplified using primers (forward: 5’ TGCTCGAGATGTGATGAAGG-3’; reverse: 5’ CTGCATTGTTTTGCCAGTGT-3’). The PCR products were separated via agarose gel electrophoresis, and images were acquired using the Gel Documentation System xR+ with Image Lab software (Bio-Rad Laboratories, Hercules, CA, USA). The amplified PCR products were quantified by analyzing band intensities using ImageJ software (NIH, Bethesda, MD, USA). The Quant-iTPicogreen dsDNA assay kit was used in parallel to independently quantify PCR products.

### Protein extraction and western blot analysis

Cells were lysed in 1× RIPA lysis buffer (Millipore # 20–188) containing protease inhibitor (Thermo Fisher Scientific, 88266) on ice for 15 min. After centrifugation at 14,000 × g for 20 min at 4 °C, the supernatants were collected and quantified using Bradford assay (Biorad, 5000006). 30 μg of each sample was resolved on NuPAGE^™^ 4 to 12%, Bis-Tris gel (Thermo Fisher Scientific, NP0322BOX) in NuPAGE^™^ MES SDS Running Buffer (Thermo Fisher Scientific, NP0002) and transferred to nitrocellulose membranes (Bio-Rad, 1620115) using the Trans-Blot Turbo Transfer Pack and System (Bio-Rad). The membranes were then blocked with TBST containing 5% skim milk for 1 h, followed by incubation with various primary antibodies (1:500–1:1000) overnight at 4 °C. Incubation with peroxidase-conjugated secondary antibodies (Donkey anti-rabbit IgG secondary antibody [Cytiva, NA934, 1:1000]; Sheep anti-mouse IgG secondary antibody [GE Healthcare, NA9310v]; donkey anti-goat IgG secondary antibody [SouthrenBiotech-6420–05, 1:1000]) was done for 1 h at room temperature. The signals were visualized using enhanced chemiluminescence (Li-cor-926–9500) and scanned using the gel documentation system (Li-Cor). Band intensity was calculated using ImageJ (NIH).

### Human ICH patient tissue collection and processing

ICH brain tissues were obtained from Houston Methodist Hospital Surgical Pathology from 3 patients during craniotomy and hematoma evacuation procedures. These patients included 2 males and 1 female, diagnosed with intraparenchymal hematoma, vascular malformation with hemorrhage, and intracerebral hemorrhage respectively. Their ages ranged from 30 to 70 years, with an average age of 54.66 years. Hematoma volumes ranged from 40 to 70 mL. Non-ICH brain tissue samples used as controls were obtained from the Binghamton Biospecimen Archive, NY, USA. The demographic and clinical details of patients and controls are provided in Supplementary Table 1.

The harvested tissue was preserved in 10% formalin and embedded in paraffin for subsequent imaging procedures. Institutional Review Board (IRB) approval (IRB number: PRO00017821) was obtained for the collection and analysis of the patient tissue samples and informed consent was obtained from all participants or their legal representatives.

### Immunohistochemistry (IHC) – TUNNEL staining

TUNEL staining was employed to assess DNA damage. Briefly, following deparaffinization and dehydration, paraffin sections were subjected to TUNEL staining using an ab206386 kit (Abcam, Cambridge, MA, USA) as per the manufacturer’s guidelines. Photomicrographs were captured using an AXIO Observer inverted microscope (Carl Zeiss, Oberkochen, Germany).

### IF microscopy of tissue sections

Human tissue was initially fixed in formalin. Subsequently, 5μm-thick paraffin-embedded sections underwent a series of steps, including deparaffinization, rehydration, and antigen retrieval in IHC Antigen Retrieval solution at 60°C for 1 h. The sections were then treated with Triton X-100 for permeabilization, followed by blocking with serum. They were incubated with either β-Gal staining solution and/or the primary antibody overnight at 4 °C. After washing in TBST, the sections were exposed to fluorophore-conjugated secondary antibodies for 60 min at room temperature and stained with DAPI. Imaging was conducted using an olympus confocal microscope fv3000 (Shinjuku City, Tokyo, Japan).

### Statistical analysis

Statistical analysis was conducted to assess the significance of the presented data. Each set of data was derived from a minimum of three independent experiments to ensure reliability and reproducibility. GraphPad Prism software was utilized for conducting the statistical analysis.

To determine significant differences within the datasets, student’s t-tests were used to evaluate to compare the means of two groups. Any p-values below the threshold of 0.05 were considered statistically significant results and the exact p values are indicated in each figure panel. A p >0.05 was considered non-significant (ns).

## Results

### Hemin treatment induces a sequential cellular response involving DDR, senescence, and HO-1 expression

Our previous studies have demonstrated that exposing neurons to hemin results in the rapid formation of DSBs in the genome and cellular senescence ^24^. Hemin treatment also induces the expression of HO-1 ^34^, but its relationship with DDR signaling and senescence remain unclear. To determine the sequence of hemin-mediated cellular events, we performed a time-course analysis using human induced pluripotent cell (iPSC)-derived neuronal cells, SH-SY5Y cells, and endothelial cells, following treatment with 10μM hemin for various time points.

**Fig. 1:**
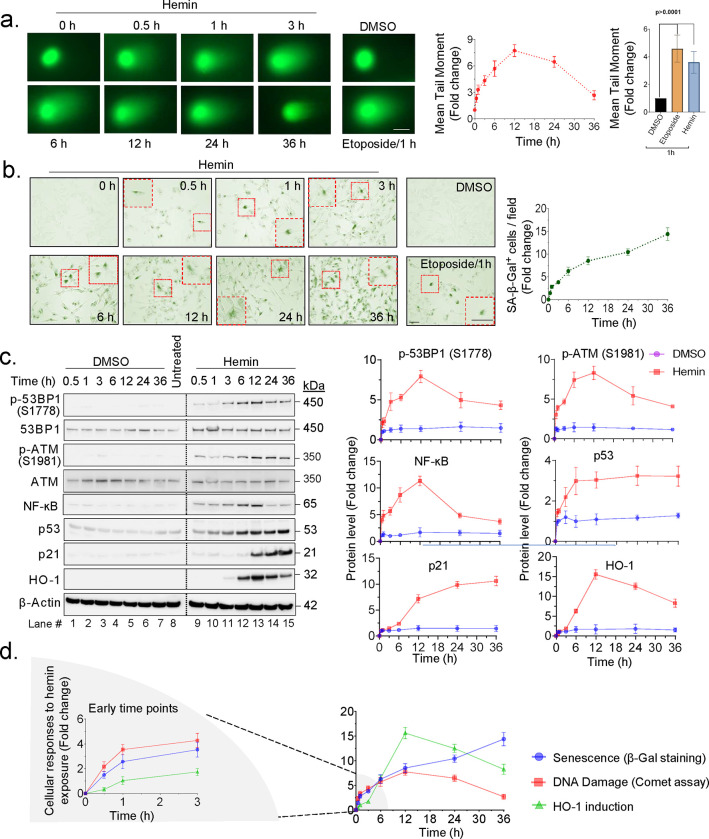
Hemin treatment leads to sequential induction of DDR, senescence, and HO-1 expression. SH-SY5Y cells were treated with hemin (10 μM) over different time intervals to analyze time-dependent kinetics, DMSO-treated cells were used as control, and etoposide (10 μM) treatment for 1 h served as a positive control. (a) A neutral comet assay was performed at the indicated time points, and the mean tail moment (fold change) of 25 randomly selected hemin-treated cells was quantified and represented in a line graph. The bar graph represents the mean tail moment fold change between DMSO, etoposide, and hemin-treated for 1 h, derived from 25 randomly selected cells, the data presented as mean ± s.e.m. from three independent experiments. (b) SA-β-Gal staining was performed to identify senescence-positive cells, representative images, and quantitation of senescent-positive cells per field in hemin-treated cells at indicated time points, the data presented is derived from three independent experiments. Scale bar = 20 μm. Zoomed insets on the red-bordered box represent 1.5x enlarged senescence-positive cells. (c) Western blot analysis of indicated DDR markers (p-53BP1-S1778, p-ATM - S1981, and p53), inflammatory marker (NF-ĸB-p65), senescence marker (p21), and heme catabolizing enzyme (HO-1) was performed on cells treated with either DMSO (lanes 1–7) or hemin (lanes 9–15) at indicated time points; untreated in lane 8. Normalized western blot band intensity (relative to representative loading control) was quantified and represented as a fold change of protein level in histograms. Total ATM and 53BP1 were used to normalize p-ATM and p-53BP1 levels respectively, and β-actin was used to normalize other proteins. The data are presented as mean ± s.e.m. from three independent experiments. (d) Time kinetics of hemin-induced cellular events such as Senescence (SA- β-Gal staining), DNA damage (comet assay), and HO-1 induction (western blot) are presented as fold change. The graph on the left shows early time points, while the graph on the right includes all the time points in the study. All statistical analyses were performed by two-sided student’s t-test, p-values are indicated in the respective graphs.

To test this, we first subjected SH-SY5Y cells to the neutral comet assay after treatment with hemin or solvent (DMSO) alone for 0.5 to 36 h. The DSB-inducing drug etoposide was used as a positive control of DDR signaling. A rapid and significant increase in DNA strand breaks was observed at 0.5 h, which is consistent with our earlier report^24^. The level of DNA damage gradually increased from 0.5 to 12 h following the treatment but then decreased at 24 h and 36 h (Fig. 1a). At 1 h, the level of DNA damage induced by 10 μM hemin was comparable to that induced by 20 μM Etoposide (Fig. 1a).

To further investigate the time-dependent activation of DDR factors such as p-ATM (S1981), p-53BP1, and p53 in hemin-treated cells, we performed Western blotting analyses. The results revealed significant DNA damage and DDR signaling as early as 0.5 h, which peaked at 12 h, gradually decreasing over time, but remaining significantly high compared to the respective only DMSO treated group, even at 36 h, consistent with the comet assay results (Fig. 1c and Supplementary Fig. 1a). We used antibodies against unmodified total proteins ATM and 53BP1 to calculate the ratio of p-ATM (S1981) to ATM (~9-fold) and p53BP1 (S1778) to 53BP1 (~8-fold) (Fig. 1c). The expression level of p53, a DDR factor, exhibited a delayed response compared to p-ATM and p-53BP1 but maintained its increased expression level steadily from 6 h to later time points post-hemin treatment. The p65 submit of the inflammatory factor NF-kB displayed a similar time-course pattern to that of p53 up to 12 h after hemin treatment. However, it then decreased at 24 and 36 h (Fig. 1c and Supplementary Fig. 1a).

To compare the time course of DDR signaling with the expression of senescence-associated β-Gal activity, we used a Senescence-β-Galactosidase Staining kit (cell signaling). The hemin-treated cells exhibited an early expression of β-Gal at 0.5 h, which increased in a time-dependent manner to a ~10-fold higher positivity compared to the only DMSO-treated cells at 12 h post-hemin treatment (Fig. 1b). The number of β-Gal-positive cells then gradually decreased after 36 h (Supplementary Fig. 1b).

**Fig. 2:**
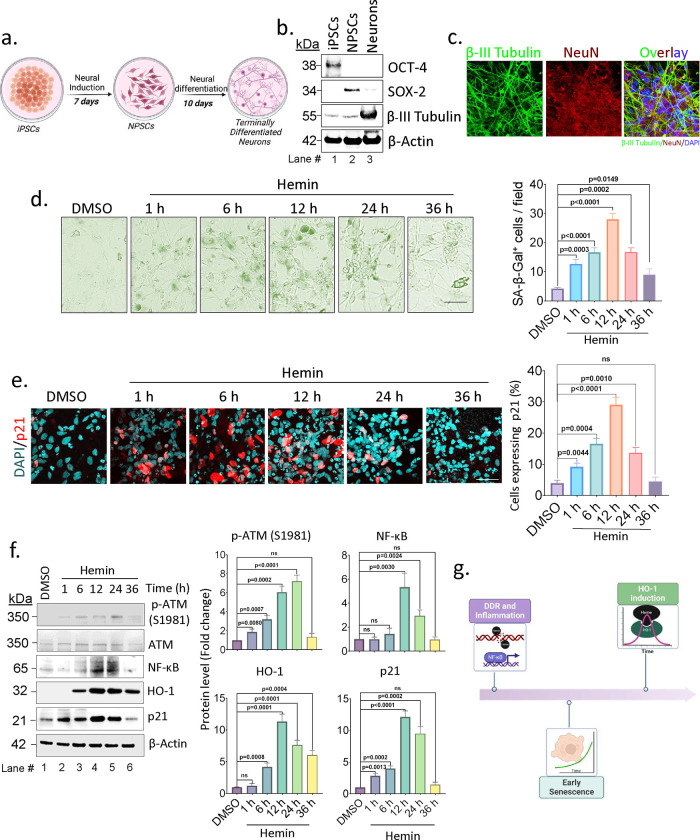
Hemin-mediated cellular events in terminally differentiated neurons. (a) A schematic representation depicting the process of differentiating neurons from iPSCs. (b) Western blot analysis of markers for iPSCs (OCT-4), NPSCs (SOX-2), and neurons (β-III Tubulin). (c) Immunofluorescence (IF) staining of iPSCs-derived neurons, showing labeling with β-III tubulin and NeuN, nuclear counterstained with DAPI. Scale bar = 10 μm. (d) Time kinetics of SA-β-Gal staining in iPSCs-derived neurons treated with hemin (10 μM), DMSO-treated cells were used as the control. Scale bar = 10 μm. Quantitation data presented as mean ± s.e.m. from three independent experiments. (e) IF analysis of p21 expression following treatment with or without hemin over time. Quantitation data presented as mean ± s.e.m. from three independent experiments. Scale bar = 20 μm. (f) Western blot analysis of p-ATM (S1981), NF-ĸB (p65), p21, and HO-1 in response to hemin treatment (lanes 2–6) at different time points. Quantitation data presented as mean ± s.e.m. from three independent experiments. (g) A schematic representation summarizing the various cellular events mediated by hemin in SH-SY5Y cells and iPSCs-derived neurons. All statistical analyses were performed by two-sided student’s t-test, p-values are indicated in the respective graph.

The time course of HO-1 expression after hemin treatment with DDR and β-Gal expression revealed that HO-1 induction occurred later than DNA damage and β-Gal expression, appearing at 3 to 6 h after hemin treatment. Its expression continued to increase up to 12 h post which was ~15-fold higher compared to the DMSO-treated control (Figs. 1c, 2f, Supplementary Figs. 1a and 1c).

We compared the pattern of hemin-induced DDR, senescence, and HO-1 expression in IPSCs derived neurons (Fig. 2d, e and f), differentiated SH-SY5Y cells (Supplementary Figs. 1c and 1d) as well as brain endothelial cells (Supplementary Figs. 2a-c) at selected time points after hemin treatment. The results showed a similar sequential cellular response in all three cell lines.

Furthermore, we examined the expression p21, a well-studied senescence marker, that has been shown to influence cell cycle arrest in dividing cells. We observed a delayed increase (at 6 to 12 h post hemin treatment) in p21 expression in SH-SY5Y cells (Figs. 1c, Supplementary Figs. 1a and 1c). This is unlike in cultured iPSCs-derived neurons where early onset of p21 expression was observed (Figs. 2e and 2f).

In conclusion, our time-course analyses of cellular responses following hemin treatment show that DNA damage and DDR signaling occur very early within 1 h, closely followed by robust β-Gal expression and then induction of HO-1 expression at 3 to 6 h. The sequential pattern of cellular response to hemin exposure is schematically shown in (Fig. 2g).

### Inhibition of DDR prevents hemin-mediated senescence and HO-1 induction

We next investigated whether hemin-induced DNA damage directly contributes to senescence-like phenotype and HO-1 induction and evaluated the effect of inhibiting the DDR pathway on these cellular events. To inhibit DDR signaling, we used transactivation inhibitors of ATM and p53 (KU-55933 and Pifithrin-α, respectively at 10 μM concentration) and evaluated the senescence status by measuring SA-β-Gal activity assay and p21 expression in hemin-treated cells at two different time points (6 and 12 h).

**Fig. 3:**
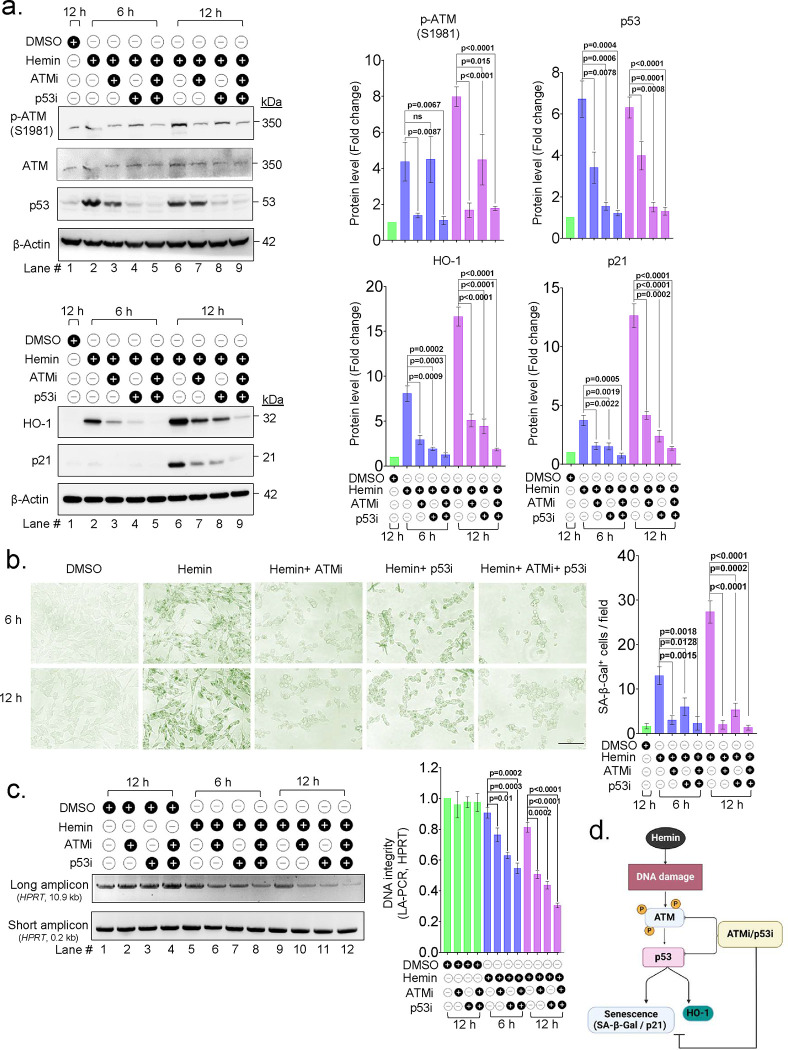
Inhibition of DDR prevents hemin-mediated senescence and HO-1 induction. SH-SY5Y cells were pre-treated with either KU 55933 (ATMi) (10 μM), Pifithrin-α (p53i) (10 μM) and in combination for 2 h before exposing them to hemin (10 μM). (a) Western blot analysis was conducted to assess the expression of p-ATM/ATM, p53, HO-1, and p21 proteins at indicated time points after hemin treatment, the band intensity was quantified and normalized using respective loading control. The quantitation data is presented as mean ± s.e.m. from three independent experiments. (b) SA-β-Gal staining was performed on cells treated with hemin and inhibitors (ATMi and p53i) at indicated time points, representative images, and quantitation of senescent positive cells per field in hemin-treated cells at indicated time points, the data presented is derived from three independent experiments. Scale bar = 20 μm. (c) DNA integrity was analyzed using LA-PCR, left panel represents an agarose gel (1%) image of the amplicon, the right panel shows the quantitation of amplified DNA using plate reader-based Picogreen assay, and represents DNA integrity (fold change) mean ± s.e.m. from three independent experiments. (d) A scheme illustrating the role of DDR factors ATM and p53 in hemin-mediated senescence and HO-1 induction. All statistical analyses were performed by two-sided student’s t-test, p-values are indicated in the respective graph.

The results showed that pre-treating the cells with the DDR pathway inhibitors prior to the hemin treatment significantly diminished the number of senescence-positive cells that exhibited β-Gal activity (Fig. 3b). Individual inhibition of ATM, p53, or their combined treatment, showed significant, but comparable levels of reduction (5 to 12%) in the senescence-expressing cell population compared to hemin alone (Fig. 3b). However, treating these DDR inhibitors alone without hemin had no effect on senescence induction (Supplementary Fig. 3a). The p21 expression in the presence of DDR pathway inhibitors showed a significant decrease compared to hemin-treated cells, like β-Gal activities (Fig. 3a). HO-1 induction was also found to be correlated with DDR signaling and blocking the DDR pathway in hemin-treated cells showed a significant decrease in the HO-1 expression compared to only hemin-treated cells (Fig. 3a).

Long-Amplification PCR analysis showed that the experimental group comprising pharmacological inhibitors ATMi or P53i together with hemin exhibited more DNA damage compared to only the hemin-treated group (Fig. 3c). These data suggested that induction of a senescence-like state and HO-1 expression in hemin-treated cells were dependent on DDR signaling. Furthermore, preventing senescence and HO-1 induction by blocking the DDR pathway after hemin treatment showed a substantial increase in cytotoxicity and a decrease in cell viability Supplementary Fig. 3b). These findings indicate that senescence state may be important in preventing acute cell death under hemin and iron-induced stress conditions.

### NF-κB-mediated transcriptional regulation of HO-1 in response to hemin

**Fig. 4:**
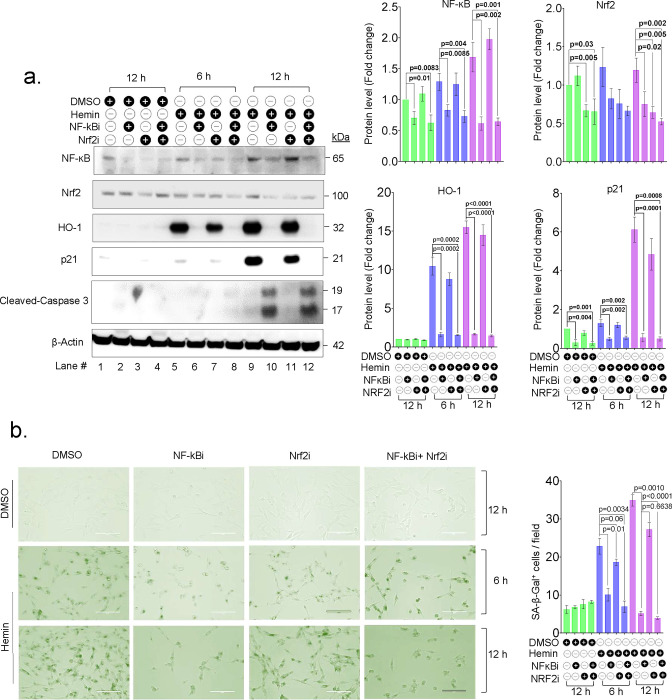
Hemin-mediated HO-1 induction is controlled by NF-ĸB. SH-SY5Y cells were pre-treated with either BMS-345541 (NF-ĸBi, 10 μM), ML385 (Nrf2i, 10 μM) or in combination for 2 h before exposure to hemin (10 μM). (a) Western blot analysis was conducted to assess the expression of NF-ĸB (p65), Nrf2, HO-1, p21, and cleaved caspase-3 proteins at indicated time points after hemin treatment, The band intensity was quantified and normalized using a respective loading control. The quantitation data is presented as mean ± s.e.m. from three independent experiments. (b) SA-β-Gal staining was performed on cells after treatment with hemin and inhibitors of NF-ĸB and Nrf2 at indicated time points representative images, and quantitation of senescent positive cells per field in hemin-treated cells at indicated time points, the data presented is derived from three independent experiments. Scale bar = 20 μm. All statistical analyses were performed by two-sided student’s t-test, p-values are indicated in the respective graph.

To explore the role of transcriptional regulators of HO-1 and their involvement in senescence induction in response to hemin treatment, we utilized pharmacological inhibitors to block the activity of two known transcription factors of HO-1, namely Nrf2 (inhibited by ML385) and NF-κB (inhibited by BMS345541). We tested the impact of blocking these transcription factors in the presence or absence of hemin. Our results showed that when NF-κB was inhibited in the presence of hemin, there was a significant downregulation of HO-1 expression. However, blocking Nrf2 and treating with hemin did not show any significant difference in HO-1 expression compared to only hemin-treated cells (Fig. 4a). These findings suggest that NF-κB plays a crucial role in hemin-induced HO-1 expression.

Furthermore, the cells treated with NF-κB inhibitor and hemin showed fewer senescence-positive cells (Fig. 4b). In addition, the cells treated with NF-κB inhibitor and hemin showed decreased p21 expression and increased expression of cleaved caspase-3 (Fig. 4a) while blocking Nrf2 did not exhibit any difference. These results indicate that NF-κB, a known anti-apoptotic transcription factor, may play a role in maintaining senescence and preventing acute cell death.

### HO-1 is co-expressed in senescence cells induced by hemin

**Fig. 5:**
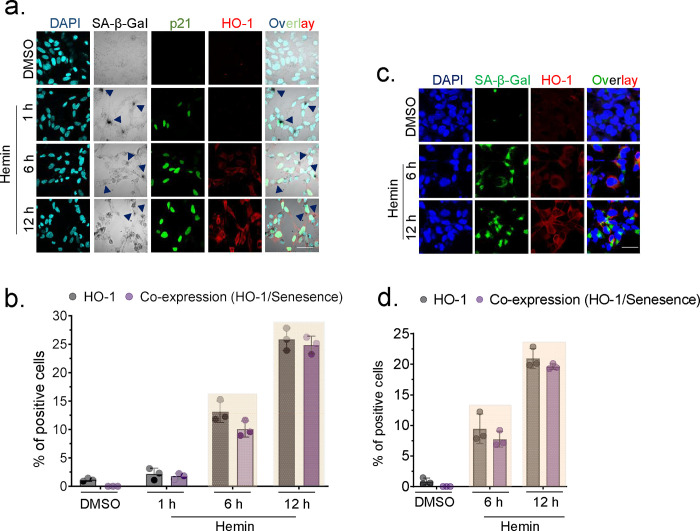
Expression of HO-1 in senescence-positive cells post hemin treatment. SH-SY5Y cells were treated with hemin (10 μM) for indicated time points, and DMSO-treated cells were used as the control. (a) Co-staining of colorimetric SA-β-Gal staining, p21, and HO-1 by IF in cells treated for 1 h, 6 h, and 12 h with 10 μM hemin. Scale bar = 20 μm. The quantitation data is presented as mean ± s.e.m. from three independent experiments, arrow indicates senescence-positive cells. (b) The co-expression of HO-1 in senescence cells (SA-β-Gal/p21) was illustrated using a grouped bar graph. (c) IF co-staining of SA-β-Gal and HO-1 was performed in cells treated with hemin for 6 h and 12 h. Scale bar = 20 μm. The quantitation data is presented as mean ± s.e.m. from three independent experiments. (d) The co-expression of HO-1 in senescence cells (SA-β-Gal) was illustrated using a grouped bar graph. All statistical analyses were performed by two-sided student’s t-test, p-values are indicated in the respective graph.

The time kinetics study shown in Fig. 1 revealed that the initial reaction to hemin-induced DNA damage in cells was the expression of senescence, with SA-β-Gal positive cells becoming evident in a short timeframe ranging from 30 min to 1 h. Subsequently, there was a more gradual increase in the expression of HO-1, occurring within a period of 3 to 6 h. To understand the relationship between senescence induction and HO-1 expression, we co-stained colorimetric SA-β-Gal with another senescence marker, p21, and HO-1. We found that cells treated with hemin exhibited SA-β-Gal and p21-positive cells at the early time point (1 h), but there was no induction of HO-1 expression. However, at the later time point (6 h), we observed increased SA-β-Gal and p21 positive cells, and HO-1 expression was induced predominantly in senescent cells but not in non-senescent cells. This phenomenon was consistent with increased expression of HO-1 in senescence positive cells at the later time point (Fig. 5a and b). We conducted a further examination of HO-1 co-expression in senescent cells by utilizing a fluorescent method to detect SA-β-Gal. This investigation was carried out at specific time points (6 and 12 h) following hemin treatment, using appropriate antibody control (Fig. 5c–d and Supplementary Fig. 4a). We observed a pattern consistent with colorimetric methods. Our results show that HO-1 expresses primarily in senescence cells post hemin exposure.

### Neuronal senescence, co-expression with HO-1 and genome damage in human ICH patient tissue

**Fig. 6:**
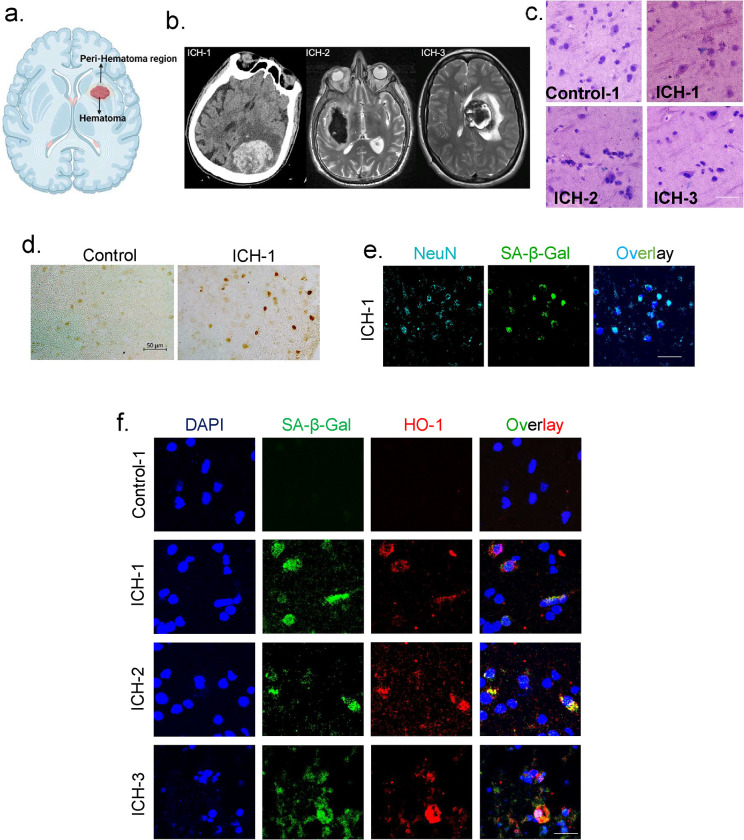
Neuronal senescence-like phenotype, co-expression with HO-1, and genome damage in human ICH patient tissue. (a) Graphical representation of the presence of hematoma and the tissue surrounding the hematoma. (b) Presurgical brain imaging of ICH patients, ICH-1: A non-contrast-enhanced computed tomography (CT) scan of the brain reveals a significant subcortical hemorrhage within the parietal lobe, measuring 5.5 × 6.4 cm. Associated findings include extensive vasogenic edema and a prominent mass effect. Hypertensive etiology was clinically correlated. ICH-2: Magnetic resonance imaging (MRI) shows large right parenchymal hemorrhage, measuring 6.8 × 2.8 × 4.5 cm. Associated findings include moderate vasogenic edema and mild midline shift. hypertensive etiology was clinically correlated. ICH-3: MRI of the brain axial T2 image shows a left parenchymal hemorrhage within the left thalamocapsular region measuring 4.5 × 3.9 × 4.6 cm in the context of recurrent cavernoma. (c) H & E staining of ICH patient tissues with appropriate age-matched non-hemorrhagic control tissue. Scale bar = 10 μm. (d) Representative image depicting DNA damage in ICH patient tissues was evaluated using the TUNEL assay compared with appropriate age-matched non-hemorrhagic controls. Scale bar = 50 μm. (e) IF co-staining of SA-β-Gal and NeuN (neuronal marker) in ICH patient tissue, Scale bar = 20 μm. (f) Immunofluorescence co-staining of SA-β-Gal and HO-1 was conducted in ICH patients (n=3), including appropriate age-matched non-hemorrhagic control (n=3), Scale bar = 20 μm.

We further extended our investigation to include brain tissue samples collected during the surgical treatment of ICH cases caused by either hypertension or vascular malformation. Our assessments included DNA damage, senescence expression, and HO-1 induction. Detailed patient information is provided in Fig. 6a and Supplementary Table 1. The tissue samples were collected following incidental removal during hematoma evacuation that was performed within 24–72 h after patient presentation. These specimens consisted of blood-exposed brain tissue immediately surrounding the hematoma, as depicted in Fig. 6a. CT/MRI images of the patient’s brain demonstrate both the presence and location of hematoma (Fig. 6b). Age-matched non-hemorrhage and non-neurological control brain samples were used for comparison (patient and control subjects’ details in Supplementary Table 1).

H&E staining was performed to represent the tissue integrity in the perihematomal region of ICH patients (Fig. 6c). TUNEL-IHC staining revealed an increase in genome damage in ICH tissues compared to the control groups (Fig. 6d). Co-staining of cells with the neuronal marker NeuN and SA-β-Gal indicated the presence of senescence expression within neuronal cells (Fig. 6e). Additionally, co-staining of SA-β-Gal and HO-1 revealed a substantial overlap in their immunofluorescence staining patterns, further validated with appropriate antibody controls (Fig. 6f and Supplementary Fig. 4b). These results illustrate the presence of a senescence-like phenotype in neurons surrounding hematomas in human ICH patients, which appear to co-localize with HO-1 expression.

### HO-1 induction in senescence cells is a crucial mechanism to prevent acute cell death post hemin treatment

**Fig. 7:**
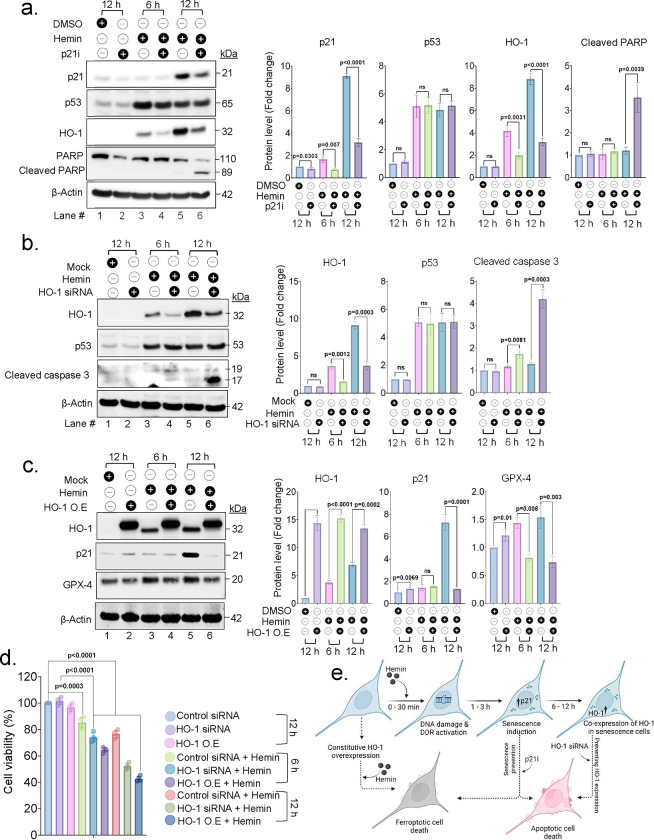
Delayed induction of HO-1 in early senescence cells crucial defense mechanism in hemin toxicity. (a) Western blot analysis was conducted with or without p21i to examine the expression of p21, p53, HO-1, and cleaved PARP proteins at the specified time points after hemin (10 μM) treatment. The quantitation data presented as mean ± s.e.m. from three independent experiments. (b) Western blot analysis was performed with or without HO-1 knockdown to examine the expression of HO-1, p53, and cleaved caspase-3 proteins at the indicated time points after hemin treatment. The quantitation data presented as mean ± s.e.m. from three independent experiments. (c) Western blot analysis was carried out after transfecting with or without HO-1 expression plasmid to examine the expression of HO-1, p21, and GPX-4 proteins at the indicated time points after hemin treatment. The quantitation data presented as mean ± s.e.m. from three independent experiments. (d) Viability assay using CellTiter-Glo reagent showing results for control cells, HO-1 knockdown cells, and HO-1 overexpressing cells at indicated time points after hemin treatment. The quantitation data presented as mean ± s.e.m. from three independent experiments. (e) A schematic illustration of the sequential cellular responses to hemin exposure and its significance in counteracting ICH-associated hemin and iron toxicity. Any interference with these sequential processes, such as inhibition of transient senescence or altering the timing of HO-1 induction without corresponding co-expression with senescence, can lead to unsynchronized cellular response and cause ferroptotic and/or apoptotic cell death. All statistical analyses were performed by two-sided student’s t-test, p-values are indicated in the respective graph.

Expression of HO-1 primarily in SA-β-Gal positive cells prompted us to explore the potential protective role of senescence during hemin degradation. To this end, senescence induction was blocked by preating cells with a known pharmacological inhibitor of p21 (UC2288), and then treated with hemin for two time points (6 and 12 h). Suppressing senescence with p21 inhibitor during hemin exposure, led to a decreased expression of HO-1 compared to the group treated with hemin alone, suggesting that HO-1 induction in senescence cells is part of a well-regulated process. Furthermore, the treatment group with p21 inhibitor and hemin showed increased expression of cleaved PARP at the 12-h time point (Fig. 7a), indicating increased apoptotic cell death, likely from hemin-induced DSB stress. The stable levels of increased p53 with or without p21 inhibitor confirms the presence of active DDR. The control group treated with hemin alone did not exhibit cleaved PARP expression. Increased cell death after hemin exposure without senescence induction suggest that senescence may offer protection against acute cell death, consistent with previous reports ^25^.

To elucidate contributions of inducible HO-1 expression after hemin treatment, we examined cell responses under two distinct conditions: first, by blocking HO-1 induction using siRNA, and second, by overexpressing HO-1. In cells treated with HO-1 siRNA and hemin, there was no significant change in p53 expression, however, there was an increased cleaved caspase-3 expression (Fig. 7b, Ln 6), indicating apoptotic cell death. Conversely, constitutive HO-1 expression using stable cells overexpressing HO-1, showed a significant decrease in GPX-4 expression (Fig. 7c). Decreased GPX-4 has been associated with propensity to ferroptosis^35^, here we can likely attribute to iron accumulation from HO-1 mediated hemin degradation prior to senescence induction. When exposed to hemin, these cells demonstrated a significant decrease in p21 expression (Fig. 7c, Ln 6), particularly at the 12-hour time point, consistent with reduced senescence in cells undergoing ferroptosis. Furthermore, analysis of cell viability showed a significant decrease in viability of both HO-1 knockdown and constitutively overexpressing cells treated with hemin compared to control cells (Fig. 7d), further affirming the crucial role of inducible HO-1 expression in senescent cells potentially for counteracting hemin and iron toxicity. We propose that interference with these sequential processes, such as inhibition of transient senescence or altering the timing of HO-1 induction, without corresponding senescence phenotype co-expression, led to unsynchronized cellular responses and caused ferroptotic and/or apoptotic cell death as illustrated in Fig. 7e.

### Prolonged exposure to hemin leads to long-lasting senescence

**Fig. 8:**
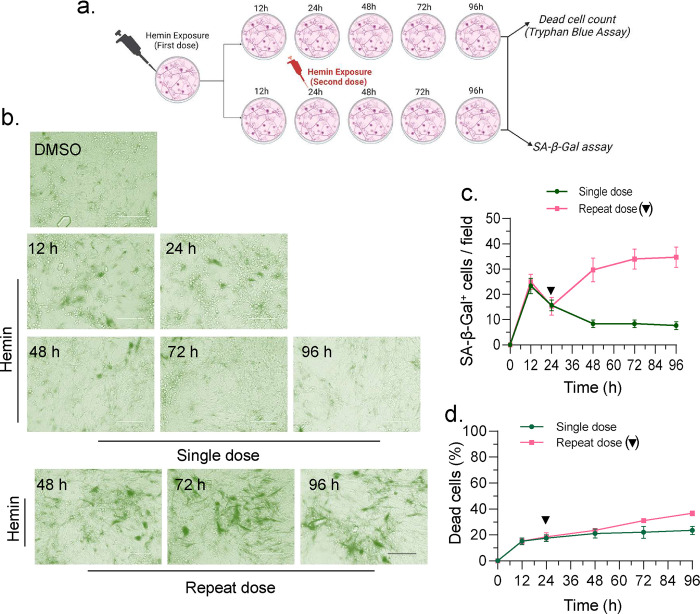
Prolonged exposure to hemin leads to long-lasting induction of senescence. (a) A schematic diagram illustrating the treatment plan of iPSCs-derived neurons with hemin, followed by subsequent experiments. (b) SA-β-Gal staining of iPSCs-derived neurons treated with or without hemin at the indicated time points. Scale bar = 20 μm. (c) Quantitation of senescence-positive cells per field in hemin-treated cells at indicated time points, arrow indicates the second dose hemin treatment time point, the data presented is derived from three independent experiments. (d) Quantification of dead cells post-hemin treatment was conducted using a trypan blue dye exclusion assay, arrow indicating the second dose hemin treatment time point.

To understand the mechanism underlying the transition from transient to prolonged senescence in ICH, we tested the effects of single versus repeat hemin treatments using iPSCs-derived neurons (Fig. 8a) or non-cycling SH-SY5Y cells (Supplementary figure 5b). Employing non-cycling conditions allowed us to evaluate the reversibility of early senescence without the potential confounding variable of cell number increase.

Initially, we treated cells with a single dose of 10 μM hemin and measured cytotoxicity (Fig. 8d) as well as senescence expression (Fig. 8b and Supplementary figure 5b) up to 96 h. This treatment resulted in increased senescence positive cells at 12 and 36 h time points in iPSCs-derived neurons or non-cycling SH-SY5Y cells respectively, as revealed by SA-β-Gal staining (Fig. 8b, 8c and Supplementary Fig. 5b and 5c). However, the senescence positivity reduced substantially to ~10% at 24 and 48-h time point in iPSCs-derived neurons or non-cycling SH-SY5Y cells (Fig. 8b, 8c and Supplementary Fig. 5b and 5c). Cell cytotoxicity data indicated that this decrease in senescence staining was not due to cell death, showing less than ~20% cell death at 48 h post-hemin treatment (Fig. 8b). These results supported the hypothesis that the early onset of senescence-like phenotype after hemin treatment is both reversible and transient.

To probe the effects of prolonged hemin exposure on senescence reversibility, we conducted a repeat hemin treatment after 36 h of the first hemin dose. The second hemin treatment resulted in a moderate increase (~35%) in cell cytotoxicity, compared to ~20% in single treatment group at 96 h (Fig. 8b). SA-β-Gal staining after repeat hemin treatment revealed a progressive increase positivity beyond 24h and 36 h (upto the analyzed 96 h time point), unlike the single treatment, in both iPSCs-derived neurons (Fig. 8b and 8c) and non-dividing SH-SY5Y cells (Supplementary Fig. 5b and 5c). Western blotting of single and repeat hemin treated cell extracts (Supplementary Fig. 5d) revealed that while HO-1 expression decreased beyond 36 h in single treatment group, a continuous upregulation of HO-1 expression was observed up to 96 h in the repeat treatment group. There was a decrease in p21 expression at later time points, and additional hemin exposure did not result in increased expression. Notably, p16, a marker associated with permanent senescence^36^, showed increased expression. These results suggest that a single moderate hemin exposure induces a transient and reversible senescence state, but prolonged hemin exposure leads to long-lasting senescence.

### Combining senescence prevention with iron chelation through bifunctional nanoparticles proves effective against hemin and iron toxicity

**Fig. 9:**
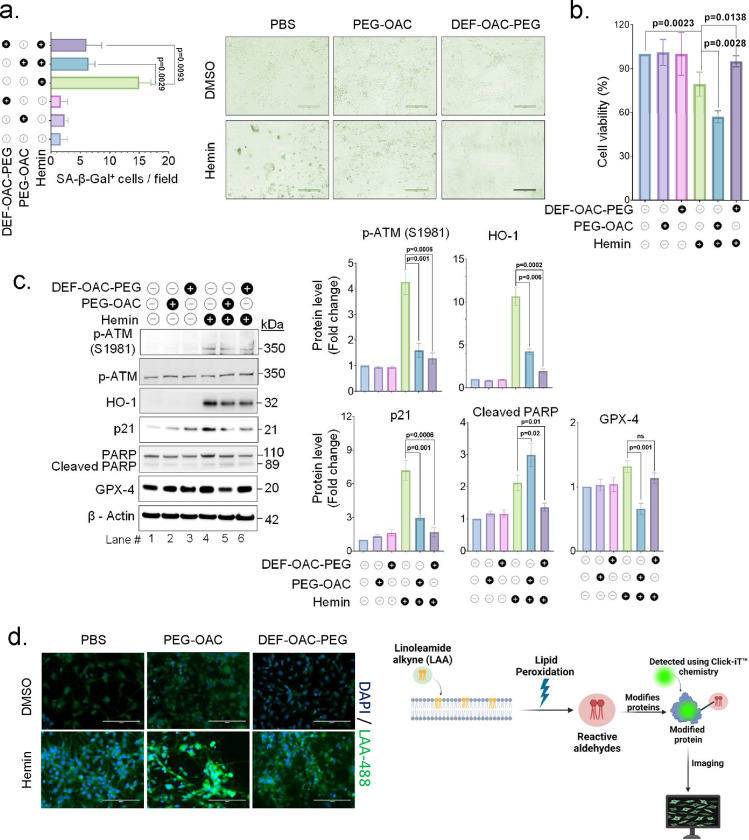
Senescence prevention by anti-oxidant nanoparticle PEG-OAC is effective only when combined with iron chelator, DEF-OAC-PEG. iPSCs-derived neurons were exposed to PEG-OAC and DEF-OAC-PEG nanoparticles 1 h after hemin (10 μM) treatment. (a) Representative images and quantification of SA-β-Gal positive cells per field in iPSCs-derived neurons treated with or without hemin and nanoparticles (PEG-OAC and DEF-OAC-PEG) for 24 h, Scale bar = 20 μm. The data presented is derived from three independent experiments. (b) Viability assay (CellTiter-Glo) of iPSCs-derived neurons treated with or without hemin and nanoparticles (PEG-OAC and DEF-OAC-PEG) for 24 h, the data presented is derived from three independent experiments. (c) Western blot analysis to assess the expression of p-ATM (S1981), HO-1, p21, cleaved PARP, and GPX-4 proteins at indicated time points, the data presented is derived from three independent experiments. (d) Representative image of lipid peroxidation assay using Click-iT^™^ lipid peroxidation imaging kit after 24 h treatment with or without hemin and nanoparticles (PEG-OAC and DEF-OAC-PEG). Scale bar = 20 μm, along with a schematic representation of the assay principle. All statistical analyses were performed by two-sided student’s t-test, p-values are indicated in the respective graph.

Building on our prior research that demonstrated the efficacy of a combined pleiotropic nanozyme and iron chelator in preventing cell death in hemin-exposed cells^24^, here we investigate additional mechanistic insights into how such combinatorial treatments interacted with the intricate cellular responses elicited by hemin exposure. We adjusted the treatable dose of PEG-OAC by experimenting with various concentrations (4, 8, and 12 μg/ml) on cells pre-treated with or without hemin. Our observations revealed a dose-responsive effect of PEG-OAC on cells treated with hemin, characterized by a decrease in the expression of p-ATM, HO-1, p21, p53, p-p65, and an increase in cleaved PARP expression (Supplementary Fig. 6). From these findings, we determined that 8 μg/ml is the most effective concentration for PEG-OAC for more in-depth subsequent research. We tested PEG-OAC and DEF-OAC-PEG in IPSCs derived neurons. The experimental group treated with either PEG-OAC or DEF-OAC-PEG nanoparticles following hemin treatment exhibited a significant reduction in the SA-β-Gal positive cells in comparison with the hemin only treated group (Fig. 9a and Supplementary Fig. 7a). However, administering PEG-OAC by itself, despite its therapeutic benefits in non-hemorrhagic brain injuries^37^ and reduction in the senescence phenotype (see below), had an adverse effect on the viability of cells exposed to hemin. In contrast, introducing deferoxamine-conjugated nanoparticles improved cellular viability (Fig. 9b and Supplementary Fig. 7b). Treating the cells with either PEG-OAC or DEF-OAC-PEG without hemin had no significant effect on cell viability in comparison to the untreated control group, whereas hemin only treated cells showed a reduction in viability (Fig. 9b and Supplementary Fig. 7b).

To decipher the molecular mechanism underlying this phenomenon, we conducted a Western blot experiment with similar experimental treatments. The findings revealed that treatment of hemin-exposed cells with either PEG-OAC or DEF-OAC-PEG led to a reduction in the DNA damage response and senescence phenotype, as confirmed by decreased p-ATM expression, decreased expression of HO-1, and senescence marker p21 (Fig. 9c and Supplementary Fig. 7c). However, cells treated with both hemin and PEG-OAC showed an increase in cleaved PARP expression, suggesting the induction of apoptosis, and a decrease in GPX-4 expression. GPX-4 is an inhibitor of ferroptosis, and thus reduction in its levels is considered as a surrogate marker indicating increase in ferroptotic cell death^38^ (Fig. 9c and Supplementary Fig. 7c). In contrast, DEF-OAC-PEG nanoparticles resulted in decreased cleaved PARP expression and increased GPX-4 expression. Taken together, these data indicate that treatment with only antioxidant PEG-OAC nanoparticles addresses DNA damage response and senescence induction but demonstrated increased cell sensitivity to apoptosis as well as ferroptosis (Fig. 9c and Supplementary Fig. 7c). A lipid peroxidation based ferroptosis assay indicated increased ferroptosis in PEG-OAC-treated but not DEF-OAC-PEG-treated cells post hemin exposure (Fig. 9d and Supplementary Fig. 7d), further corroborating the need for addressing iron mediated ferroptosis with senescence prevention in ICH conditions.

In summary, our results demonstrate that hemin induces DNA damage, triggering a transient senescence state in neurons, with delayed expression of the hemin-degrading enzyme HO-1. Repeated hemin exposure transitions this senescence to a long-lasting state, which can be prevented by inhibiting DNA damage response or suppressing p21, which however, ultimately leads to increased ferroptotic cell death likely due to the presence of free iron that also accumulates during ICH. Combining senescence prevention with iron chelation using a tailored nanoparticle effectively mitigates hemin-induced senescence and protects against ferroptosis, unveiling potential strategies to address both senescence and iron toxicity in brain hemorrhage.

## Discussion

The findings presented in this study shed light on cellular mechanisms underlying *in vitro* model of brain hemorrhage with human samples correlation. Our results demonstrate that transient senescence, induced via the DDR, reduces apoptotic as well as ferroptotic cell death in the short term, but with prolonged exposure results in persistent senescence, that may ultimately result in longer term detrimental effects on brain function^39, 40, 41, 42, 43^.

**Fig. 10:**
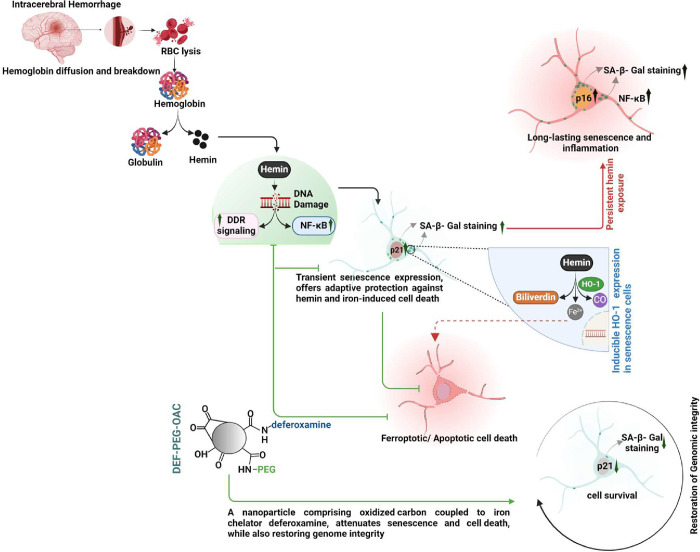
A model depicting the mechanism of cellular response to hemin exposure. Intracerebral hemolysis results in the release of hemin from ruptured blood cells. Initially, cells respond by activating DNA damage response (DDR) and NF-ĸB pathways, leading to a temporary onset of the senescence like phenotype. This response acts as a critical protective mechanism against the neurotoxic effects of hemin and iron following a brain hemorrhage. This is followed by the stimulation of HO-1, an enzyme responsible for breaking down heme. However, continuous exposure to hemin can cause cells to enter a state of prolonged senescence. Our study demonstrates that a bifunctional carbon nanoparticle comprising anti-oxidant + iron chelating activity alleviates genome damage and cellular senescence.

A key observation in our study is the temporal relationship between DDR, senescence, and HO-1 induction in response to hemin exposure and the importance of these sequential events in managing hemin toxicity. Time course analysis revealed rapid and early induction of DSBs and DDR signaling within 30 min of hemin treatment, suggesting cellular recognition of hemin-induced genotoxic stress. This early DDR signaling is followed by the expression of senescence-like phenotype, as evidenced by SA-β-Gal staining. Importantly, we observed a pre-senescence state, triggered directly by DDR signaling and characterized by its transient nature. The direct role of DDR in mediating cellular responses to hemin was demonstrated by inhibiting DDR signaling using pharmacological inhibitors of ATM and p53, which resulted in reduced senescence leading to increased cytotoxicity. HO-1 induction then occurs predominantly in these pre-senescence cells, demonstrated by nearly exclusive co-localization of HO-1 and senescence-associated SA-β-Gal staining in hemin-treated cultured cells as well as ICH patient tissue. This reversible senescence phase likely served as a temporary protective mechanism adapted by the cells against iron toxicity, until free iron generated by HO-1 mediated degradation of hemin is bound by storage proteins like ferritin^26^. Increased cell death via ferroptosis when senescence is prevented by inhibiting DDR or p21 supports its potential protective role against iron toxicity. Transient senescence thus may offer cells enhanced capacity to manage acute iron concentrations, thereby impeding cell death pathways such as ferroptosis. Moreover, our investigation revealed the reversible nature of early senescent response to hemin treatment, contrasting with the progression to irreversible senescence observed with prolonged exposure to hemin, characterized by p16 expression, a marker associated with irreversible senescence^44^. This progression to long-lasting senescence may be associated with cellular aging and neurological dysfunction, highlighting the nuanced impact of hemin/hemoglobin on cells^45^. This nuanced understanding of protective versus pathological roles of senescence induction has implications for the reversibility of senescence-related processes in the context of hemin exposure, as schematically shown Fig. 10. These findings provide first experimental evidence that cells exposed to low-dose hemin undergo transient senescence, a process that may provide an adaptive protective function in ICH and blood toxicity conditions.

The importance of sequential expression of DDR-mediated senescence and HO-1 expression in senescence cells for cell survival post hemin treatment was further demonstrated by overexpression of HO-1 prior to hemin treatment, which resulted in increased ferroptosis, in the absence of transient senescence expression. As expected, inhibiting HO-1 expression also leads to increased cell death. Transcriptional regulation of HO-1 and senescence in response to hemin was also explored, with a focus on the involvement of the transcription factor NF-κB. Inhibition of NF-κB led to downregulated HO-1 expression, reduced senescence, and increased apoptotic cell death, highlighting the significance of NF-κB in the cellular response to hemin.

Notably, we observed a comparable pattern of hemin-mediated DDR, HO-1 induction, and senescence-like phenotype across various cell types, including cultured neurons and endothelial cells. While our study primarily focused on cultured cells, we acknowledge there is complexity of *in vivo* responses to hemin, influenced by the proximity of cells to hematoma^46^. We previously confirmed that subcortical injection of hemolyzed blood in mice increased expression in the peri-hematoma region of the DDR-related protein, gamma H_2_AX, paralleling that seen here in culture^24^. Importantly, here our findings were validated in human ICH patient tissue samples, confirming the presence of genome damage, senescence expression, and HO-1 induction in neurons surrounding hematomas. Crucially, as in cultured cells exposed to hemin, HO-1 expression was observed primarily in β-Gal positive senescence cells in ICH tissue. Furthermore, there was a substantial co-localization of SA-β-Gal staining with NeuN positivity, suggesting the expression of senescence-like phenotype in human brain neurons. This clinical validation underscores the relevance and consistency of the identified cellular responses across different experimental systems and clinical contexts.

Our study supports a potential adaptive role of transient senescence state as a neuroprotective pathway in response to genotoxic stress induced by hemin and iron. Consequently, strategies aimed at preventing senescence or removing senescent cells should be carefully evaluated in hemin-related disorders, with consideration for their combination with iron chelators. This integrated approach, as demonstrated by the efficacy of bifunctional nanoparticles DEF-OAC-PEG, offers promising avenues for the management of conditions associated with hemin exposure.

Limitations of our study include reliance on common senescence markers such as SA-β-Gal staining, p21, and p16 and the need for comprehensive metabolic and transcriptomic profiling to better understand the dynamic states of senescence. However, our results demonstrating DDR-dependence of the observed SA-β-Gal activity suggests is indicative of senescence. A comparative evaluation of hemin-mediated senescence and HO-1 expression in other CNS cell types (e.g., astrocytes and microglia) is a subject for future investigation. While blood also contains other sources of toxicity including hemoglobin itself and thrombin^47, 48^, which may involve additional mechanisms of injury.

In conclusion, our study provides a comprehensive mechanistic understanding of cellular responses to hemin exposure in the context of brain hemorrhage, highlighting the sequential activation of DDR, senescence, and HO-1 induction as critical mechanisms for cell survival and confirms many of these signals from *in vitro* studies are observed in human samples. The study also reveals a crucial balance between the protective role of transient senescence in ICH and their eventual progression to a long-lasting senescence state that may be associated with cellular aging and neurological dysfunction^39, 40^. Altogether, these findings offer insights into the development of DDR and senescence-targeted therapeutic strategies for managing hemin and iron toxicity in hemin-related disorders, with implications for improving clinical outcomes.
